# Choline and Cystine Deficient Diets in Animal Models with Hepatocellular Injury: Evaluation of Oxidative Stress and Expression of RAGE, TNF-*α*, and IL-1*β*

**DOI:** 10.1155/2015/121925

**Published:** 2015-06-02

**Authors:** Juliana Célia F. Santos, Orlando R. P. de Araújo, Iara B. Valentim, Kívia Queiroz de Andrade, Fabiana Andréa Moura, Salete Smaniotto, John Marques dos Santos, Juciano Gasparotto, Daniel P. Gelain, Marília O. F. Goulart

**Affiliations:** ^1^Faculdade de Nutrição, Universidade Federal de Alagoas (UFAL), Campus A. C. Simões, Avenida Lourival Melo Mota, S/N, Tabuleiro dos Martins, 57072-970 Maceió, AL, Brazil; ^2^Instituto de Química e Biotecnologia (IQB), Universidade Federal de Alagoas (UFAL), Avenida Lourival Melo Mota, s/n, Cidade Universitária, 57072-900 Maceió, AL, Brazil; ^3^Instituto Federal de Educação, Ciência e Tecnologia de Alagoas, 57020-600 Maceió, AL, Brazil; ^4^Programa de Pós Graduação em Ciências da Saúde (PPGCS), Universidade Federal de Alagoas (UFAL), Campus A. C. Simões, Avenida Lourival Melo Mota, s/n, Tabuleiro dos Martins, 57072-970 Maceió, AL, Brazil; ^5^Instituto de Ciências Biológicas e da Saúde, Universidade Federal de Alagoas, 57072-900 Maceió, AL, Brazil; ^6^Centro de Estudos em Estresse Oxidativo (CEEO), Departamento de Bioquímica, Instituto de Ciências Básicas da Saúde (ICBS), Universidade Federal do Rio Grande do Sul (UFRGS), Rua Ramiro Barcelos 2600 Anexo, Santana, 90035003 Porto Alegre, RS, Brazil; ^7^INCT de Bioanalítica, UNICAMP, 13083-861 Campinas, SP, Brazil

## Abstract

This study aims to evaluate the effects of diets deficient in choline and/or cystine on hepatocellular injury in animal models (young male Wistar rats, aged 21 days), by monitoring some of the oxidative stress biomarkers and the expression of RAGE, TNF-*α*, and IL-1*β*. The animals were divided into 6 groups (*n* = 10) and submitted to different diets over 30 days: AIN-93 diet (standard, St), AIN-93 choline deficient (CD) diet and AIN-93 choline and cystine deficient (CCD) diet, in the pellet (pl) and powder (pw) diet forms. Independently of the diet form, AIN-93 diet already led to hepatic steatosis and CD/CCD diets provoked hepatic damage. The increase of lipid peroxidation, represented by the evaluation of thiobarbituric acid reactive species, associated with the decrease of levels of antioxidant enzymes, were the parameters with higher significance toward redox profile in this model of hepatic injury. Regarding inflammation, in relation to TNF-*α*, higher levels were evidenced in CD_(pl)_, while, for IL-1*β*, no significant alteration was detected. RAGE expression was practically the same in all groups, with exception of CCD_(pw)_ versus CCD_(pl)_. These results together confirm that AIN-93 causes hepatic steatosis and choline and/or cysteine deficiencies produce important hepatic injury associated with oxidative stress and inflammatory profiles.

## 1. Introduction

Hepatic steatosis (HS) is a growing clinical problem worldwide. It is associated with obesity, insulin resistance (IR), diabetes mellitus (DM), and metabolic syndrome (MS) and is one of the alterations that characterize nonalcoholic fatty liver disease (NAFLD) [1–4]. The estimated world prevalence of NAFLD, based on various evaluation methods, ranges from 6.3 to 33% of the population, with an average of 20%. The main risk factors for the disease are obesity, type 2 diabetes, and dyslipidemia. It is estimated that there are at least 1.46 billion obese adults worldwide, from which 6 million people will progress to nonalcoholic steatohepatitis (NASH) and some 600,000 to NASH-related cirrhosis [3–7].

Several experimental models have been used to elucidate the mechanisms of NAFLD etiology (histologically categorized into HS and NASH), particularly using diets low in the lipotropic factor choline and in methionine [6, 8]. Methionine is an amino acid inserted into the cycle of choline, cysteine/cystine, and glutathione. Absence or dietary deficiency of any of these compounds would favor hepatic triglyceride accumulation, due to defective formation of very low density lipoproteins (VLDL) and reduced *β*-oxidation of lipids. In addition, this deficiency leads to the committal of the methionine transsulfuration via the diminution of glutathione levels, thus reducing important part of the intracellular antioxidant defense [4, 5, 8, 9].

The liver contains a potent enzymatic and nonenzymatic antioxidant defense system. Liver cells, especially hepatocytes, have a substantial capacity to metabolize and effectively detoxify harmful species, such as reactive oxygen and nitrogen species (ROS/RNS). However, in the presence of liver injury of any kind, these functions suffer significant drawbacks [10].

Oxidative stress (OS), which reflects an imbalance between prooxidants and antioxidants with increased reactive oxygen species (ROS) and/or decrease of antioxidants, is characteristic of several diseases (acute or chronic) that affect the liver. It includes induction of inflammatory process* via* Kupffer and Ito cells activation, monocytes, macrophages, and dendritic cells as well as natural killer cell recruitment. In this step, these cells recruited in response to injury emit proinflammatory signals including cytokines (TNF-*α*, IL-6, and IL-1), chemokines, lipid messengers, and ROS. This results in a moderate self-limiting inflammation or advancement of apoptosis or necrosis, which aggravates the inflammatory response and may lead to fibrosis [11–13].

Another pathway of inducing OS and liver inflammation occurs through the production of advanced glycation end products (AGEs). AGEs are a heterogeneous group of molecules produced by* in vivo* glycation and oxidation that modify chemical and biological properties of several molecules [14, 15]. AGEs are also formed exogenously by heating foods at high temperature and low humidity and could enhance, together with endogenous AGE, the dangerous action into the body [16]. Depending on the cell type and experimental conditions, AGEs interaction with their receptors, RAGE, in hepatocytes and hepatic stellate cells leads to an increased production of ROS through activation of nicotinamide adenine dinucleotide phosphate (NADPH) oxidase. This enhances cell proliferation and activation, thus playing a role in the progression of hepatic fibrosis to NASH [14, 17, 18].

There are many questions regarding the pathophysiological mechanism of liver diseases. A variety of dietary treatments can be used to induce liver damage; however the model of lipotropic factors deficient diets induces a more aggressive damage with the rapid progress toward NASH [6, 8]. In this study, AIN-93G (growth) diet as a standard diet and the experimental models of NAFLD were chosen, according to REEVES [19]. This formulation has casein as the standard protein and cystine and choline as adding factors. The experimental models, compared to the standard (St) diet are based on the nonaddition of these lipotropic dietary factors.

Along this line, the present paper aims to evaluate the effects of diets deficient in choline and for the first time, in choline-cystine, on hepatocellular injury in animal models by monitoring some of the OS biomarkers such as protein carbonylation and TBARS, related to oxidative damage, enzymatic antioxidant defense, characterized by catalase (CAT) and superoxide dismutase (SOD) activities, and nonenzymatic defense, represented by total thiols. In addition, the presence of inflammation through RAGE, TNF-*α*, and IL-1*β* expression was evaluated.

## 2. Material and Methods

### 2.1. Animals and Chemicals

Male* Wistar* rats (30–35 g, aged 21 days) were obtained from the laboratory animal house at the Federal University of Alagoas (UFAL), Maceio, Alagoas, Brazil. The animals were kept in individual metabolic cages in the experimental laboratory animal house of the School of Nutrition, UFAL. The animals had free access to water and food and were maintained in a 12 h light-dark cycle (lights on at 7 AM), under controlled temperature (20–24°C). These conditions were kept constant throughout the experiments. All experimental procedures were performed in accordance with the* National Institute of Health Guide for the Care and Use of Laboratory Animals* and the* Brazilian Society for Neuroscience and Behavior Recommendations for Animal Care* (006549/2011-10). All chemicals were purchased from Sigma Aldrich (St. Louis, Missouri).

### 2.2. Protocol

The animals were divided into 6 groups (*n* = 10) (Table 1) and submitted to different diets during 30 days. Diets were formulated from a food composition of the* American Institute of Nutrition* (AIN, version 93) [19] with inducers of NAFLD in the form of pellets_(pl)_ and powder_(pw)_: a standard (St) (AIN-93 in its original composition), CD (AIN-93 deficient in choline), and CCD (AIN-93 deficient in choline and cystine).

The contents of the original sucrose diet (10%) were replaced by corn starch. The diets were provided by* RHOSTER Industry and Commerce Ltd.* (São Paulo, Brazil). The body mass and food intake of the animals were measured weekly.

At the end of experimental period, after fast of 12 hours, the animals were anesthetized (ketamine, 100 mg/kg, and xylazine, 15 mg/kg, via intraperitoneal injection in the right inferior quadrant of rat) and euthanized by sectioning off the aorta. Blood was collected and the plasma separated for subsequent biochemical analysis: fasting glucose, lipid profile, and liver function and injury. The liver was dissected, placed on liquid nitrogen, and immediately stored at −80°C for later analysis. Immediately before the analysis, the organs were homogenized in PBS 10 mM with Potter-Elvehjem apparatus and centrifuged (10000 g, 10 min at 4°C) to remove cellular debris. Supernatants were used for all stress oxidative biochemical assays. Protein (ptn) content was quantified using the Lowry protein assay [20].

### 2.3. Histological Analysis

After fixation with 10% buffered formalin, the organs were cleaved and the sections were processed by embedding in paraffin and stained with hematoxylin and eosin. Macroscopic alterations of the organs were recorded whenever present. Histopathological evaluation to determine hepatocellular damage was based on the guidelines of “The Diagnosis and Management of Non-Alcoholic Fatty Liver Disease: Practice Guideline by the American Gastroenterological Association, American Association for the Study of Liver Diseases, and the American College of Gastroenterology” [3].

### 2.4. Oxidative Damage

#### 2.4.1. Thiobarbituric Acid Reactive Species

TBARS (thiobarbituric acid reactive species) is widely adopted as a method for measurement of the lipid redox state [21]. Briefly, the samples were mixed with 0.6 mL of 10% trichloroacetic acid (TCA) and 0.5 mL of 0.67% thiobarbituric acid and then heated in a boiling water bath for 25 min. TBARS was determined by spectrophotometry at 532 nm. TBARS concentration in the samples was obtained from a calibration curve that was prepared using 1,1,3,3-tetramethoxypropane (TMP) as the standard, which was subjected to the same treatment as that applied to the supernatants of the samples. Results are expressed as nmol TBARS/mg protein.

#### 2.4.2. Carbonylated Proteins

Oxidative protein damage was measured by the quantification of carbonyl groups based on their reaction with 2,4-dinitrophenylhydrazine (DNPH). Proteins were precipitated by the addition of 20% TCA and resuspended in 10 mM DNPH and the absorbance read at 370 nm [22]. Results were expressed as nmol carbonyl/mg protein.

### 2.5. Antioxidant Enzymes

Catalase (CAT) activity [23] was measured as described in the literature [24]. The rate of decrease in absorbance at 240 nm was used as an index of H_2_O_2_ degradation by catalase. One unit of CAT was considered the amount of enzyme needed to degrade 1 mmol/min H_2_O_2_ at 25°C. Superoxide dismutase (SOD) activity was assessed by quantifying the inhibition of superoxide dependent epinephrine autooxidation in a spectrophotometer at 480 nm [25]. The assay was carried out in 96-well microplates, using a Thermo Scientific UV-Vis microplate spectrophotometer.

### 2.6. Nonenzymatic Antioxidants

#### 2.6.1. Total Reduced Thiol Content

To quantify the content of reduced thiols, samples were diluted in 10 mM phosphate buffer (pH 7.4), followed by the addition of 5,5′-dithiobis(2-nitrobenzoic acid) (DTNB) (0.01 M), in ethanol. An intense yellow color was developed and read at 412 nm after 20 min. A blank control was run simultaneously, without addition of DTNB. Protein thiol content was calculated after subtraction of the blank absorbance, utilizing the molar extinction coefficient of 13 600 M/cm [26].

### 2.7. Analysis of Western Blot for RAGE, IL-1*β*, and TNF-*α* in the Hepatic Tissue

To perform immunoblot experiments, liver tissue samples were homogenized in Laemmli sample buffer (62.5 mM Tris-HCl, pH 6.8, 1% (w/v) SDS, and 10% (v/v) glycerol) and then equal amounts of the protein homogenate (40 *μ*g/well) were fractionated, quantified by SDS-PAGE, and electroblotted onto nitrocellulose membranes. Protein loading and electroblotting efficiency were verified through Ponceau S staining, and the membrane was blocked with Tween Tris buffered saline (TTBS: 100 mM Tris-HCl, pH 7.5, 0.9% NaCl, and 0.1% Tween-20) containing 5% albumin. Membranes were incubated overnight at 4°C with each antibody separately in TTBS and at different working dilutions, as suggested by the manufacturers, and then washed with TTBS. Anti-rabbit IgG peroxidase linked with a secondary antibody was incubated with the membranes for an additional 1 h (1 : 5,000 dilution range), it was washed again, and the immunoreactivity detected by enhanced chemiluminescence. Thereafter, the membranes were incubated for 12 h with anti-rabbit primary from Sigma Aldrich (anti-IL1*β* and anti-TNF-*α*, ABCAM; anti-RAGE (N-terminal)) (1 : 1,000, anti-*β*-actin dilution range, Cell Signaling) and then washed again and the immunoreactivity was detected by enhanced chemiluminescence. Densitometric analysis was carried out with ImageJ software. All results were expressed as a ratio relative to the *β*-actin internal control.

### 2.8. Statistical Analysis

Results were shown as mean ± standard error (SEM) for each group. Statistical analysis was performed using GraphPad Prism. For multiple comparisons, one-way analysis of variance (ANOVA) was used. In the case of ANOVA, the results showed significant differences. Post hoc analysis was performed with Tukey's test. *p* < 0.05 was considered to be statistically significant.

## 3. Results

### 3.1. Analysis of Body Weight and Absolute and Relative Liver Weights

The parameters of food intake, weight gain, body weight end, liver weight, and relative liver showed no statistical difference (*p* ≥ 0.05) (Table 2).

### 3.2. AIN-93 Diet (Pellets and Powder) and Its Influence on Hepatic Steatosis

**Figure 1:**
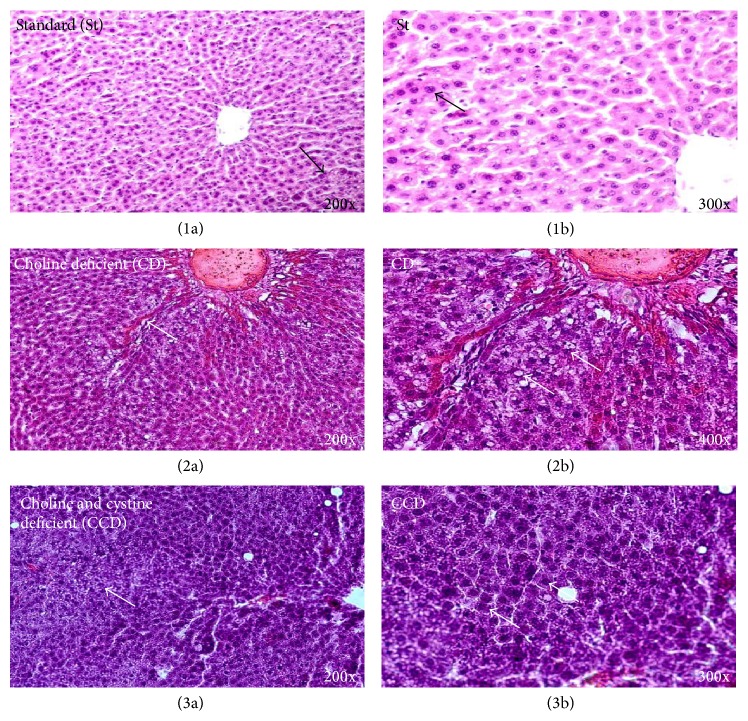
Representative photomicrographs of hematoxylin and eosin staining of liver sections. St (1a) (200x): arrow indicating periportal zone with disorganized hepatocyte cords; St (1b) (300x): hepatic steatosis, arrow indicating fat accumulation in hepatic intracellular vacuole; CD (2a) (200x): arrow indicating hepatic cord disorganisation around centrilobular zone, hepatic steatosis, and signals suggestive of fibrosis; CD (2b) (400x): arrows indicating ballooning degeneration of hepatocytes and macro and microvesicular steatosis; CCD (3a) (200x): arrow indicating hepatic cord disorganisation, nucleus with increased size, and microvesicular steatosis; St (3b) (300x): right arrow indicating nucleus with increased size and arrow nuclear breakdown, Mallory corpuscles.

Animals fed with AIN-93 diet for 30 days (St_pw/pl_) showed hepatocellular damage, independently of the absence of lipotropic factors and the diet presentation (pw or pl). The standard (St) group had mild HS, with disorganized hepatocyte cords next to the periportal zone (Figures 1(a) and 1(b)). On the other hand, the CD group showed intense HS, independently of the zone, with disorganized hepatocyte cords throughout the hepatic tissue, ballooning of hepatocytes, few inflammatory infiltrates, and cellular death (Figures 1(c) and 1(d)). In the CCD group, disorganized hepatocyte cords similar to the CD group were found, as well as microvesicular steatosis, an increase in the size of nucleus or its death in hepatocytes, Mallory corpuscles, and cellular death, while hepatocyte ballooning or inflammatory infiltrates (Figures 1(e) and 1(f)) were not found.

### 3.3. Analysis of Redox Profile

Regarding nonenzymatic analyses, in relation to TBARS, the St_(pw/pl)_ groups showed the same TBARS levels as the CD_(pw)_ group and lower than CD_(pl)_ and CCD_(pw/pl)_ groups (*p* < 0.05). CD_(pl)_ and CCD_(pw/pl)_ groups were statistically similar (*p* ≥ 0.05). Toward carbonylated protein the CD_(pw)_ group showed lower levels than the CCD_(pw/pl)_ group (*p* < 0.05). The other groups were statistically similar (*p* ≥ 0.05).

**Figure 2:**
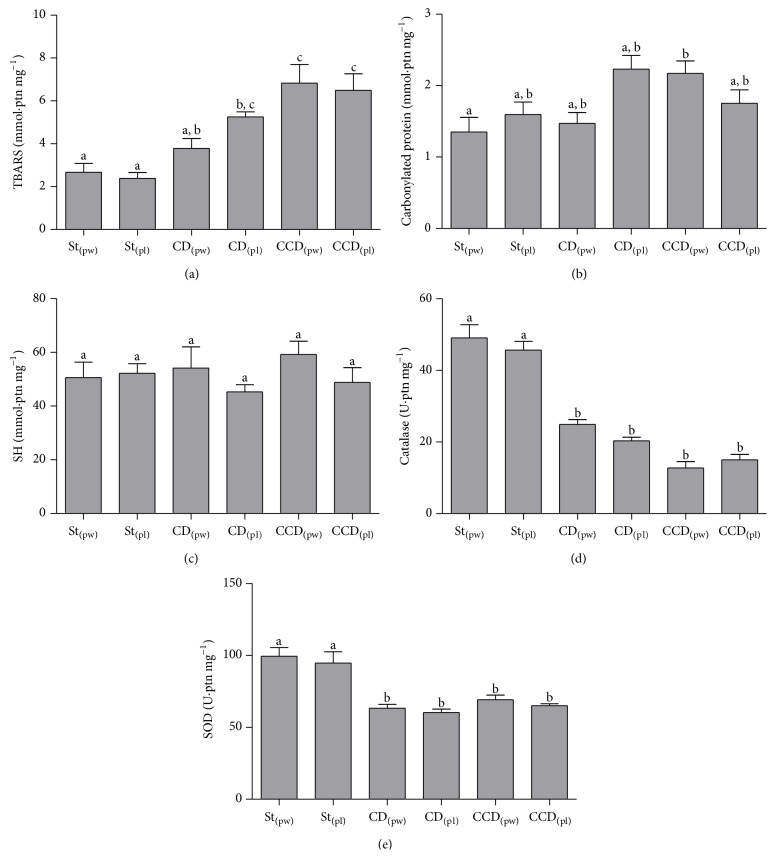
TBARS (a), carbonylated protein (b), thiols group (SH) (c), catalase (d), and SOD (e) in liver tissue for the different dietary models (mean ± SEM). Standard (St); CD: deficient in choline; CCD: deficient in choline and cystine; pl: pellet; pw: powder. Different letters indicate *p* < 0.05 (Tukey test).

In the evaluation of antioxidant enzymes, hepatic SOD and CAT activities were lower in the CD_(pw/pl)_ and CCD_(pw/pl)_ animals compared to the St_(pw/pl)_ (*p* < 0.05). In relation to nonenzymatic antioxidants, glutathione and other thiols were measured as total thiols. All groups fed with AIN-93 diet were statistically similar (*p* ≥ 0.05) (Figure 2).

### 3.4. Analysis of RAGE, TNF-*α*, and IL-1*β* Expression

**Figure 3:**
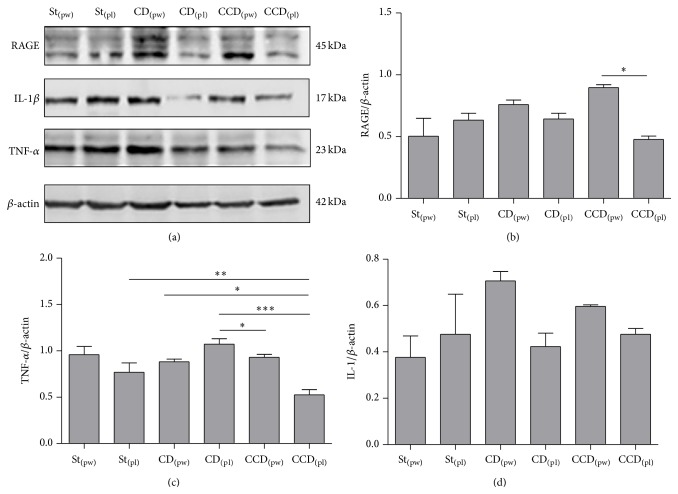
Western blot analysis of RAGE, IL-1*β*, and TNF-*α* (*β*-actin internal control) (a). Mean ± SE Protein content of RAGE (b), IL-1*β* (c), and TNF-*α* (d) in rat livers for different dietary models (mean ± SEM); Standard (St); CD: deficient in choline; CCD: deficient in choline and cystine; pl: pellet; pw: powder. ^*∗*^ *p* < 0.05; ^*∗∗*^ *p* < 0.01; ^*∗∗∗*^ *p* < 0.001 (Tukey test).

In this study, RAGE expression by immunoblot detection was highest in the CDD_(pw)_ group compared to CCD_(pl)_ (*p* < 0.05) groups and similar to other groups, all of which were statistically similar (*p* ≥ 0.05) (Figure 3(a)). In respect to TNF-*α* (Figure 3(b)), its expression was higher in the CD_(pl)_ group than the other groups (*p* < 0.05), except for the St_(pw)_ group (*p* ≥ 0.05). The CCD_(pl)_ group had the lowest levels of TNF-*α* (*P* < 0.001) between the experimental groups, except the St_(pw)_ group, which was statistically similar (*p* ≥ 0.05). In respect to IL-1*β* expression (Figure 3(d)), all groups fed with AIN-93 diet were statistically similar (*p* ≥ 0.05).

## 4. Discussion

Diets deficient in choline are related with HS development [27]. Choline is a water soluble essential nutrient (quaternary ammonium salt). Choline and its metabolites are needed for three main physiological purposes: structural integrity and signaling roles for cell membranes, cholinergic neurotransmission (acetylcholine synthesis), and being a major source for methyl groups via its metabolite, trimethylglycine (betaine), which participates in the S-adenosylmethionine (SAMe) synthesis pathways [28, 29]. As reported, choline presents several functions in the body; however it is mostly phosphorylated and used as part of membrane phospholipids or oxidized such as methyl group donor [30]. Thus, when a choline deficiency occurs, the VLDL synthesis is impaired, once this lipoprotein needs phosphatidylcholine in its structure.

Additionally, choline is part of mitochondrial membrane and its deficiency causes alterations in this organelle and consequently leads to NAFLD. It is believed that complex I is one of the major sources of ROS in liver mitochondria and, in case of choline deficiency, the liver mitochondria composition is significantly altered. This could be associated with changes in respiratory chain complex I activity, called NADH: ubiquinone oxidoreductase and this could be modulated in rats fed with choline deficient diet [30, 31].

Recently, the scientific community recommended the use of choline deficient (CD) diet, on an exclusive basis. These formulations cause NASH, but the worsening of this disease is prolonged when compared to alteration caused by a diet deficient in both choline and methionine (CMD). Additionally, CMD can promote hepatic fibrosis and hepatocellular carcinoma (HCC) [32]. Although CMD was shown to cause a more intense hepatic injury, this food restriction presents some limitations. It was reported that rats fed with CMD diet had weight loss up to 35% in 4 weeks. It was also shown that depending on the rodent species, the NAFLD induced by CMD varies considerably [27].

As an example, Veteläinen and coworkers [33] identified weight loss, severe steatosis, increased OS (evaluated by TBARS and reduced glutathione levels), decreased antioxidant capacity, and NASH progression (inflammation mediated by KC and fibrogenesis) in animals that received CMD diet for 7 weeks [33]. In contrast, mice fed with the CD diet developed uncomplicated steatosis or a profile similar to the human metabolic syndrome (MS), such as insulin resistance (IR), dyslipidemia, and obesity. These models represent two different NAFLD pathogenic forms. In our study, the diet model chosen was AIN-93M (maintenance), in which the amino acid of addition is cystine whose precursor is methionine. It is believed that* L*-cysteine has a sparing effect on methionine, in reducing the catabolism of methionine through a transsulfuration pathway [9]. Thus,* via* cystine deficient diet, methionine present in casein will be used to restore cysteine levels, causing combined deficiency of synthesis of other compounds methionine dependents, such as choline and GSH, and together those alterations are able to cause hepatic injury and oxidative stress. Thus, the results were similar to the ones earlier obtained [33], with increased hepatocellular damage and OS being identified in animals receiving a diet deficient in both lipotropic factors (CCD). As such, our model (Table 1) allowed the evaluation of NAFLD progression, as well as differences and similarities in the quantification of OS at different moments in the pathophysiological process.

However, for final body weight, absolute, and relative liver weight (Table 2), no significant differences were observed, as well as serum biochemistry (data not shown). Our hypothesis was that the experimental time was not enough for the development of such changes.

Another fact that deserves attention in this study was that animals fed with AIN-93 standard (St) diet showed mild hepatocellular damage (HS). This fact had already been identified in previous studies in different contexts [34–37], suggesting that the AIN-93 composition is inappropriate and justifying, therefore, our option for the present diet (Table 1), and which, independent of changes in diet composition, led to HS. One hypothesis to explain this liver damage is related to inadequate amounts of choline and sulfur-containing amino acids in the diet.

In respect to evaluation of oxidative damage, it has been known that carbonyl groups quantification is largely used as a marker of oxidative damage in proteins [38]. In our study, we found significant differences in the carbonylated protein analysis only between DCC_(pw)_ and DC_(pw)_ groups, with high levels of carbonyl in severe liver damage group. Besides protein damage, the membrane injury caused by lipid peroxidation (LP) is also an oxidative stress consequence [11]. In this context, it was observed that the St_(pw/pl)_ groups showed a lower TBARS level than CD_(pl)_ and CCD_(pw/pl)_ groups (*p* < 0.05). LP causes direct damage to cell membranes (and organelles), generates intermediates which can serve as a substrate for the synthesis of toxic compounds to the environment, and still produces intermediate metabolites involved in the carbonylation reaction [4]. Protein carbonylation may have contributed to the reduction in circulating levels of protein. According to several authors, modified proteins cell death persists if oxidized proteins are not removed by the proteasome [39, 40].

Simultaneously with lipid accumulation in liver, an alteration in electron transport chain can occur, causing an increase of ROS production, in terms of anion radical superoxide (O_2_^∙−^) and hydrogen peroxide (H_2_O_2_). These species can oxidize polyunsaturated fatty acids (PUFAs) present in cell and organelle membranes leading to several events and producing LP metabolites like malondialdehyde (MDA) and 4-hydroxynonenal (4-HNE) [41]. Moreover, the LP of PUFAs in mitochondrial membrane has been associated with apolipoprotein B (ApoB) proteolysis, and this reduces VLDL secretion in rodents, promoting triacylglycerol accumulation in liver [1] and causing severe morphofunctional alterations in both deficient diet groups in this study.

LP metabolites cause alteration in both deoxyribonucleic acid (DNA) and protein synthesis, decrease GSH levels, increase proinflammatory cytokines synthesis, promote influx of inflammatory cells into liver, and activate Ito cells, causing collagen deposition, hepatic fibrosis, hepatocytes death, and necrosis, all of which being histological characteristics of NAFDL evaluation [1, 42].

The antioxidant defense provided by thiols, like glutathione complex, shows no significant difference between groups, regardless of the presence of HS or severe hepatocellular damage, unlike the results found for CAT and SOD, whose activity levels decreased with the severity of liver damage. In the study by Bakala et al. [43], it was suggested that the redox cycle of glutathione was the largest source of protection in the presence of low levels of hydrogen peroxide, whereas CAT becomes more significant in cytoprotection, in severe OS, when glutathione concentration had already been depleted [43]. This fact can be explained both by protein carbonylation which decreases the activity of the sulfur components with antioxidant functions and by the dietary model used, since the reduction of lipotropic factors contributes to decrease of glutathione synthesis [44, 45]. Elevated levels of carbonylated proteins also contribute to a reduction in SOD activity, since intermediary metabolites like methylglyoxal react with lysine and arginine, components of this enzyme, and as a result there is an elevation in the levels of superoxide anion and inhibition of CAT activity [46].

OS is closely related to the inflammatory process, a condition observed in many diseases such as cardiovascular disease (CVD) [47–50], cancer [51–53], and gastrointestinal diseases [3, 54, 55]. Several markers had been used to verify inflammatory activity* in vivo*. Currently, in the scientific literature, there is emphasis on identifying RAGE expression, once this receptor has the ability to recognize multiple ligands and therefore can participate in pathophysiological events related primarily to the propagation of cell dysfunction [15, 56] and increased the synthesis of various interleukins, such as IL-6 and IL-1*β* [57–60].

The lower expression of RAGE in CCD_(pl)_ compared to CDD_(pw)_ group may indicate a decrease of inflammatory activity in the animals fed with deficient diet pellets group. Similar data were observed for TNF-*α* expression. It has suggested that the advance of liver damage in this group, demonstrated by histology, has contributed to the exhaustion of these inflammatory markers in this tissue.

**Figure 4:**
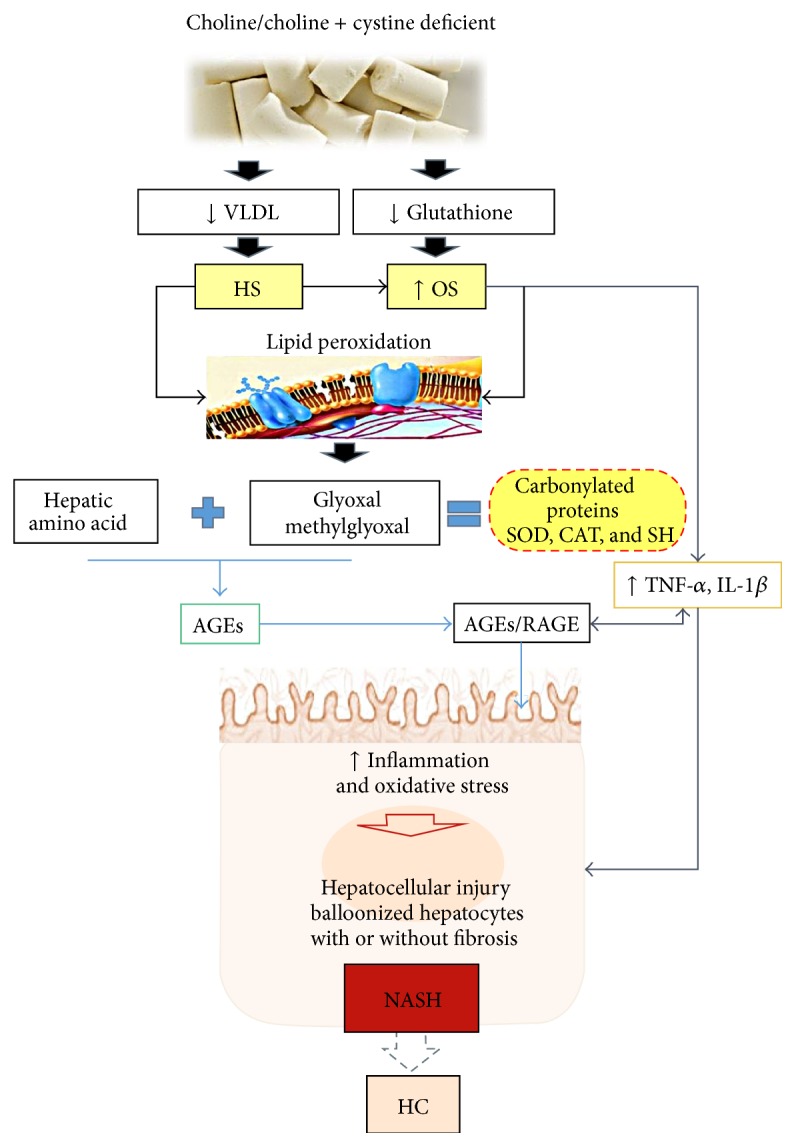
Diets deficient in choline (CD) or choline and cystine (CCD) decrease the formation of hepatic VLDL and antioxidants derived from thiols (for instance, glutathione), leading to hepatic steatosis (HS) and increased susceptibility to oxidative stress (OS). HS leads to OS and lipid peroxidation (LP) (directly); additionally, OS also stimulates the production of inflammatory cytokines. LP leads to the formation of reactive intermediates (glyoxal and methylglyoxal) which by interacting with proteins (amino acids) cause liver tissue modifications: carbonylation of proteins including enzymatic antioxidants (SOD, CAT, and SH) and formation of AGEs. AGEs can interact with cell receptors, RAGE, triggering a cascade reaction that increases inflammation and intracellular OS. Additionally, inflammatory cytokines increased directly by OS also contribute to the reaction described above, resulting in hepatocellular injury, hepatocyte ballooning with or without fibrosis, leading to NASH and eventually to hepatocellular carcinoma (HC).

It was emphasized, in our study, that HS was present in all standard groups and in the one deficient in choline, regardless of the form of the diet, if in powder or in pellets. In addition, an important fact demonstrated by the analysis of inflammatory markers was that the intensity of steatosis was not able to change the RAGE expression between the groups, demonstrating that inflammation may be present from the mild fat accumulation. An overall picture of our results is shown in Figure 4 (Figure 4).

In the study by Gaens and coworkers [61], which evaluated liver biopsies and serum samples from obese individuals, the authors observed that the immune-histochemical staining of RAGE in biopsies was specifically and exclusively located in the membrane of hepatocytes with steatosis, whereas hepatocytes without steatosis did not show staining for RAGE. Also according to these authors, lipid accumulation in hepatocytes (*in vitro*) was associated with increased expression of RAGE, plasminogen activator inhibitor-1 (PAI-1), interleukin 8 (IL-8), interleukin 6 (IL-6), and C-reactive protein (CRP), all related to the inflammatory process [61].

The study by Leung and coworkers [62] compared the expression of RAGE in three groups of animals fed with diets in pelletized form: one fed with a standard diet, another with a CMD (cysteine/methionine deficient) diet, and finally a group that was fed with a CMD diet prepared with additional heating. One of the results observed was an increase in these receptors (RAGE) in the third group, most likely due to the higher amount of exogenous AGEs ingested [62].

RAGE activation has been implicated in inflammation as well as in cancer, diabetes, and Alzheimer's disease [63]. Strong upregulation of RAGE and its ligands were found in different tumors and experimental evidence supports a critical role for RAGE and its ligands in tumorigenesis and metastasis, by still unknown mechanisms [63, 64]. Pusterla et al. [65], studying RAGE expression in oval cell from liver by two models: a genetic model of Mdr2−/− RAGE−/− double knockout mice and a pharmacologic blockade of RAGE model, found that RAGE ablation does not affect inflammatory cell recruitment; however the authors identified a novel function of RAGE in regulating oval cell activation and tumor development in inflammation-associated liver carcinogenesis [65]. Confirming these findings, Ito et al. [66], studying the expression of RAGE in hepatectomized patients, verified that the higher the expression of these receptors was, the worse the therapeutic outcome of these patients was (lower survival and further development of hepatocellular carcinoma) and, therefore, future therapeutic approaches aimed at the blocking of RAGE need to be tested [66].

Activation of RAGEs has as consequence, the stimulation of nuclear factor kappa B (NF-*κ*B) that increases the production of various proinflammatory substances, among them IL-1*β* and TNF-*α* [67]. In our study, IL-1*β*, involved in hepatic lipid accumulation, cellular death, and fibrogenesis [57, 58], did not differ between groups.

The pathogenesis of NASH involves several steps including lipotoxicity, intestinal endotoxins, and innate immune responses such as toll-like receptors (TLRs), proinflammatory cytokines, and OS in the endoplasmic reticulum. Inflammatory cytokines such as TNF-*α*, IL-6, and IL-1 may be produced by Kupffer cells, which also contribute to increased hepatic injury through complement factor synthesis and ROS [57–59].

TNF-*α* contributes to inflammation, IR, and HS, by modulating SREBP-f (sterol regulatory element-binding protein) activity. TNF-*α* may be produced by other cells besides KCs, such as adipose tissue cells and hepatocytes [4]. This factor contributes significantly to cellular dysfunction by promoting the production of ROS/RNS by causing a dysfunction in the electron transport chain, inducing cell death through the activation of caspase-8 and the apoptotic cascade in the cytosol [59].

Despite the conflicting results of this study for TNF-*α*, a growing body of evidence supports a central role of TNF-*α* and other inflammatory cytokines in the progression of HS to NASH, with a correlation between circulating levels of these cytokines and the severity of steatosis, necroinflammation and fibrosis [59].

## 5. Conclusions

The CD/CCD diets were sufficient to provoke liver damage, regardless of the form (powder or pellets) they were given in, allowing the differentiation or discrimination of the various evolutionary stages of NAFLD. Moreover, for all the models studied, the powder or pellet forms did not lead to significant differences in any of the evaluated parameters. Another important fact was the presence of HS in rats fed with the AIN-93 diet in its standard formulation, demonstrating that this food, for this study, was unsuitable for animal feed. Another noteworthy point was the study of the antioxidant compounds, where the thiol groups represented the first system of defense to be consumed, with a reduction in their levels from mild hepatocellular damage, unlike the enzymatic compounds evaluated.

Regards to inflammation, TNF-*α* revealed a synchronicity with the data for RAGE and both of these with the redox imbalance data.

However, further studies are necessary for a better understanding of the advanced stages of the disease, as well as the expression of inflammation markers.

## Figures and Tables

**Table 1:** AIN-93 formulation according to selected groups.

Group	Diet formulation
Standard St_(pw)_/St_(pl)_	AIN-93

Choline deficient CD_(pw)_/CD_(pl)_	AIN-93 deficient in choline: powder (pw) or pellet (pl)

Choline and cystine Deficient CCD_(pw)_/CD_(pl)_	AIN-93 deficient in choline and cystine: powder (pw) or pellet (pl)

**Table 2:** Food intake, weight gain, end weight of body, liver weight, and relative liver weight for the different dietary models (mean ± SEM). Standard: St; CD: deficient in choline; CCD: deficient in choline and cystine; pl: pellet; pw: powder.

	St_(pw)_	St_(pl)_	CD_(pw)_	CD_(pl)_	CCD_(pw)_	CCD_(pl)_
Food intake (g)	102.70 ± 5.00^a^	104.00 ± 5.24^a^	97.51 ± 5.07^a^	101.80 ± 5.31^a^	103.70 ± 4.319^a^	100.30 ± 5.60^a^
Weight gain (g)	145.20 ± 3.55^a^	157.50 ± 5.53^a^	145.90 ± 7.29^a^	154.80 ± 5.90^a^	143.70 ± 4.40^a^	155.2 ± 6.87^a^
End weight of body (g)	176.80 ± 3.59^a^	190.00 ± 5.85^a^	177.10 ± 7.54^a^	185.90 ± 5.69^a^	175.90 ± 5.34^a^	188.30 ± 7.73^a^
Liver weight (g)	7.39 ± 0.31^a^	7.65 ± 0.33^a^	7.46 ± 0.31^a^	7.65 ± 0.28^a^	7.42 ± 0.22^a^	7.55 ± 0.30^a^
Relative liver weight	0.04 ± 0.00^a^	0.04 ± 0.00^a^	0.04 ± 0.00^a^	0.04 ± 0.00^a^	0.04 ± 0.00^a^	0.04 ± 0.00^a^

^a^Means similarity with other a, in terms of statistics. Different letters indicate *p* < 0.05 (Tukey test).
